# Needs and Self-Care Efficacy for Cancer Patients Suffering from Side Effects of Chemotherapy

**DOI:** 10.1155/2021/8880366

**Published:** 2021-04-24

**Authors:** Sae'd Abu El-Kass, Marwa M. Ragheb, Safaa' M. Hamed, Anas M. Turkman, Azhar T. Zaki

**Affiliations:** ^1^Department of Nursing, Faculty of Medical Sciences, Al Aqsa University, Gaza Strip, State of Palestine; ^2^Department of Nursing and Health Sciences, University College of Applied Sciences, Gaza Strip, State of Palestine; ^3^Department of Medical Surgical Nursing, Faculty of Nursing, Benha University, Qalyubia, Egypt; ^4^Faculty of Nursing, Benha University, Qalyubia, Egypt; ^5^Vice Dean for Environmental Development and Community Service, Professor of Medical Surgical Nursing Department, Faculty of Nursing, Benha University, Qalyubia, Egypt; ^6^Medical Surgical Nursing Department, Faculty of Nursing, Benha University, Qalyubia, Egypt; ^7^Mental Health Hospital, Department of In-service Education, Ministry of Health Arar, Arar, Saudi Arabia; ^8^Technical Nursing Institute, Faculty of Nursing, Ain Shams University, Cairo, Egypt

## Abstract

*Background and Aim*. Cancer is the leading cause of death in economically developed countries and is a threat to human lives. Cancer and chemotherapy side effects may affect the daily activity of cancer patients and their families on many levels confronted by changes in health status and lifestyles, leading to impaired self-care efficacy. *Objective*. To assess the needs and self-care efficacy for cancer patients suffering from side effects of chemotherapy. A descriptive cross-sectional design was conducted. A purposive sample of 150 adult cancer patients undergoing chemotherapy during the period from January to June 2020 was studied at the Oncology Outpatients Clinic at Al Rantisi Hospital in Gaza Strip. *Tools*. Tools of the study involved the following: structure interviewing questionnaire, patient assessment needs' tool, and self-care activity for side effects of chemotherapy; Part I: assessment of self-care efficacy and Part II: assessment of physical, psychological, social, and spiritual needs of patients and activity of daily living. The findings of this study indicated that, concerning the duration of illness, 44.5% of studied patients started complaining of symptoms of cancer for about two years, and more than one-third of them, 69.3%, started chemotherapy more than one year ago. More than half of the studied patients, 55.3%, had poor knowledge about cancer, side effects of chemotherapy, how to manage these side effects, and level of self-care efficacy. The majority of studied subjects, 87.3%, had a financial burden, and nearly two-thirds of patients, 61.3%, need reassurance to cope with illness. There was high statistical significance between self-care efficacy and daily living activity. Regarding physical problem, the most affected systems were the gastrointestinal and the dermatological system. Also, the majority of patients were independent in walking, dressing, toileting, and feeding, but more than two-thirds of them needed assistance toward the ability to handle finance, shopping, housekeeping, food preparation, and travelling.

## 1. Introduction

Cancer is a major health problem in Palestine, as well as the world. Cancer is one of the leading causes of mortality worldwide. Approximately 18 million new cases are discovered annually, and 9.6 million mortality cases are reported worldwide due to cancer, according to global cancer statistics in 2018 [1].

Chemotherapy is the systematic treatment of cancer with anticancer agents. It works by stopping the growth of cancer cells, which abnormally grow and divide quickly. Unfortunately, it cannot selectively distinguish between healthy cells and cancer cells. Chemotherapy induced side effects are a result of hurt to healthy cells [2].

Self-Care refers to the activities, individuals, their families, their communities, and others to enhancing health and maintain good physical and psychological well-being, preventing disease, and maintain health and wellbeing after illness or discharge from hospital [3]. Self-care refers to decisions and actions that an individual can take to cope with a health problem or to improve his or her health [4].

However, persons undergoing chemotherapy might alter their self-care practices in order to meet the physiological and psychological wellness after treatment. Therefore, the nurse has a role in identifying the quantity and quality of self-care deficits in patients with cancer and providing the knowledge, skills, and support necessary to cope with their illness [5].

Cancer is the second leading cause of death, after heart disease, with 14.7% of deaths in Palestine [6]. Therefore, the mortality rate is 128.9 per 100,000 population for the 20–60 age group, representing 22.1% of all deaths [6].

It was observed that a large number of cancer patients were admitted to the chemotherapy clinic and oncology department. Those patients have a lack of knowledge about appropriate self-care activities. The aim of this study was to assess needs and self-care efficacy for cancer patients who suffer from side effects of chemotherapy.

## 2. Materials and Methods

### 2.1. Study Design and Setting

This present study is a descriptive analytic cross-sectional study. The cross-sectional study will help the researcher to take a snapshot of the current health problem under investigation. The study was conducted at the Outpatient Clinic of Oncology at Al Rantisi Specialized Hospital in Gaza Strip from January to June 2020. The study was conducted among adult cancer patients undergoing chemotherapy at the Outpatient Clinic of Oncology at Al Rantisi Specialized Hospital.

### 2.2. Study Sample and Sampling

A purposive sample was applied to select the required participants. The present study included “150” patients with cancer undergoing chemotherapy aged from 20 to 60 years old, from both genders, regardless of their cancer types and cancer stages during the time period from the beginning of January to the end of June 2020 and agreed to participate in our study voluntarily.

### 2.3. Study Instrument

Sociodemographic data were used to describe characteristics of patients under study regarding age, gender, educational level, occupation, income, cost of treatment, and marital status, “ten questions.” It includes questions to assess patient's knowledge about the disease, the definition of cancer, causes of cancer, the purpose of chemotherapy, various methods of administration of chemotherapy, and side effects of chemotherapy, “nine questions” Smeltzer et al. [5]. The score of the patient's knowledge about cancer and chemotherapy treatment was assayed by questions in the form of a rating scale based on “3” points: It ranges from “0” that indicates does not know, to “2” for complete knowledge. Data was gathered through face-to-face interviews. Pilot interviews were conducted with “15” patients with cancer to ensure clarity of questions. Results of the pilot were excluded from the final data collection. Two well-trained data collectors were recruited to gather the data from the eligible cancer patients in the Oncology Clinic at Al Rantisi Specialized hospital.

Patient assessment needs' tool and self-care efficacy are based on “2” parts.

Part I: it is concerned with the assessment of self-care efficacy regarding personal hygiene, prevention of infection, how to manage side effects of chemotherapy as pain, fatigue, fever, constipation, diarrhea, hair loss, extravasation, anorexia, bleeding, mouth ulcer, and vomiting.

#### 2.3.1. Scoring System

Questions were in the form of multiple-choice questions: “true” or “false.” The responses were scored as “1” for a correct answer and “0” for an incorrect answer.

Part II: it is concerned with the assessment of physical, psychological, social, and spiritual needs of patients and activity of daily living.

The assessment of the social, psychological, and spiritual needs of patients was evaluated according to “14” items, including social interaction and financial burden by Longo et al. [7]. The assessment of psychological needs of patients was evaluated, including the difficulty of keeping their own role opinion about side effects of chemotherapy and relationship with religion Farrell and Grant [8].

Total to all questions was evaluated as follows:
    < 60% was considered poor knowledge
    ≥ 60% was considered good knowledge

The activity of daily living scale was measured using two instruments; the Basic Efficacy of Daily Living (BADLs) and the Instrumental Efficacy of Daily Living (IADLs). These instruments are modified from the studies of El Hamed Ali et al. [9] and Carpenito-Moyet [10].

^∗^(ADLs): it deals with the efficacy of daily life by using Katz Index [11], to assess the physical needs of cancer patients. It was adapted and modified by the researcher based on Glacken et al. [12] and Black, Hawks, and Keene [13] to assess the physical needs of patients regarding daily living activity according to “6” items “feeding, bathing, toileting, transferring, walking, travelling, and dressing.”

^∗^(IADLs): the Lawton Instrumental Efficacy of Daily Living IADLs Scalewas adopted and modified by the researcher based on Graf [14].

There are “6” domains of function measured with the Lawton IADL scale; women were scored on all “6” areas of function, and men were not scored in the domains of food preparation and housekeeping. However, current recommendations are to assess all domains for both genders, according to Lawton et al. [15]. People are scored according to their highest level of functioning in that category. A summary of score ranges from “0” that indicates low function, dependent, to “6” for high function, independent.

It deals with activities such as the ability to use the telephone, housekeeping, ability to handle finance, shopping, responsibility for own medication, and food preparation.

For a total scoring system in both Basic Efficacy of Daily Living and Instrumental Efficacy of Daily Living, each function is related on three points scale: It ranges from “0” that indicates totally dependent, to “2” for totally independent.

Total score for every patient was evaluated as follows:

  < 60% was considered dependent
  ≥ 60% was considered independent

### 2.4. Data Analysis

All data were collected, tabulated, and subjected to statistical analysis. Statistical Package for Social Sciences Software Version 22 is performed by Microsoft Office for data handling and graphical presentation. Quantitative variables are described by mean standard deviation, while qualitative categorical variables are described by proportions and percentages. Chi-squared test of independence is used for categorical variables. Test of significance was used, and regarding the significance of the result, the observed difference and association were considered as follows:

Nonsignificant *P* > 0.05, significant *P* ≤ 0.05, and highly significant *P* ≤ 0.01.

## 3. Results

One hundred fifty patients with cancer participated.  Table 1 shows the mean age of the studied patients was 42.67 ± 7.29, and 54.7% were females. Regarding their occupation, 64.7% of patients did not work; in relation to marital status, 77.3% of studied patients were married; concerning the education level, 32.7% of them were of average education. About 46.7% of patients have a husband or wife who takes care of them. 94.7% of patients were treated by the government, free of charge, on medical assurance. In relation to monthly income, 81.3% of patients stated that they have no sufficient income for treatment. 72.0% of studied patients had a negative family history of cancer. 95.3% of patients in the study were aware of their diagnosis. Regarding the type of cancer, about 25.3% of patients in the study had breast cancer.

Table 2 shows that 58.0% of patients did not know the type of chemotherapy received, and 69.3% of them started chemotherapy for more than one year before the study, but 81.3% of them were not feeling better after chemotherapy. The majority of studied subjects had side effects after the session with a few hours, 82.0%, while 60.7% of them were showing the side effects of chemotherapy shortly after the given dose, in the range of few days to one week. 50.7% of patients became side-effect-free about two weeks after chemotherapy.

Table 3 shows that regarding social needs, the majority of studied patients, 87.3%, 89.3%, had a financial burden; 70.0% of them had interference with caring responsibility and home activity. Concerning psychological needs, about 46.0%, 42.0% of them were feeling patient and calm and had good social interaction with family members. But the majority of studied patients, 81.3%, were not exercising in order to relieve stress “such as hot showers.” Regards the religious needs, the majority of studied subjects, 90.7%, were accepting changes in their life, and 70.7%, 79.3% of the patients were realizing the importance of participation in religious activities in order to receive spiritual support, but 50.7% of subjects have no purpose or aim in their life.

Table 4 shows that regarding the level of independence in Basic Efficacy of Daily Living (ADLs) among the study subjects revealed that more than three-quarters of patients were independent toward walking, feeding, toileting, dressing, and ability to use telephone, about 81.3%, 96.0%, 93.3%, 78,0%, 76.7% regarding advanced or Instrumental Efficacy of Daily Living (IADLs) of subjects, and about 72. 7%, 74.7%, 68.7%, 74.7%, 69.3% needed assistance to handle finance, shopping, housekeeping, food preparation, and travelling.

Table 5 shows that there are highly statistically significant differences between the patients' level of knowledge and their physical and social needs at *P* ≤ 0.001. Also, there are statistically significant differences between spiritual needs and level of knowledge at *P* ≤ 0.05. There is a nonstatistically significant relation between the level of knowledge and their psychological needs at *P* > 0.05.

Table 6 shows that, the relations between patients' level of independence and their sociodemographic variables among study subjects; there are highly statistically significant differences between the level of independence and their education level and age at *P* ≤ 0.001. Also, there are statistically significant differences between the level of independence and their marital status, job, monthly income, cost of treatment, family member, and presence of “depending-on” person at *P* ≤ 0.05. There is a nonstatistically significant difference between the level of independence and their sex at *P* > 0.05.

Table 7 shows that there were a positive correlation and high statistical significance between self-care efficacy and daily living activities.

## 4. Discussion

Cancer and its treatment are a disruptive and life-threatening process. Chemotherapy is one of the most important treatments for cancer patients, but it has many side effects and serious complications that affect the physical, psychological, social, and spiritual dimensions of an individual's life and alter their functioning in the usual efficacy for many months or even years. Therefore, patients under chemotherapy need information related to chemotherapy treatment and schedule, including information on the potential side effects, self-care needs, and the effects on work and relationships that are the key factors for successful treatment [16].

Findings of this study revealed that more than one-quarter of the studied subjects are in the age group 50–60 years. This finding may indicate that cancer was common among older age groups, and more than three-quarters of them were female. This finding is similar to reports from Asian Americans [17], who reported that the mean age of their study group was 21 to 86 years, in which the majority of them were females. This finding is supported by Jung et al. [18], which reported cancer is primarily a disease of older people, with rates increasing rapidly from around age 50 and peaking in the eldest age groups for most cancers.

More than three-fourths of the studied patients were married, and the majority of patients were not working because their health condition forced them to stop work or led them to have fewer delegated responsibilities. This finding is consistent with Couzin-Frankel [19], who reported that the majority of patients with cancer are not working or are unemployed. Another result of the present study revealed that about one-third of the patients were of average education. So, most of them were aware of their diagnosis, in line with studies' findings by Üstündağ and Zencirci [20], who stated that only ten percent of study patients with cancer were illiterate.

Related to chemotherapy, the current study showed that the majority of the studied patients did not know the type of chemotherapeutic agent received. This may be due to the neglection of the staff to share information with cancer patients about treatment plans and schedules, who may be unaware of the importance of this action to the patient, or due to the very high load of cancer cases receiving therapy in the only and central hospital in Gaza Strip for cancer, which is Al Rantisi Hospital, where there is no time for the staff to share every single piece of information with the patient, or due to absence of consent form that encourages or even forces the staff to counsel with the patient before giving chemotherapy, or due to low educational level of the majority of our patients, that made the doctor unable to discuss managing plans with them. This result is inconsistent with the National Cancer Institute [21], which assured that the doctor should put the treatment plan, including the type of chemotherapy, number and duration of sessions, and everything about their chemotherapy, with the patient.

This study revealed that more than two-thirds of patients started chemotherapy for more than one year before the study. This may be due to the selection of authors to patients who are in need of self-care education.

In our study, we found that the side effects of chemotherapy appear shortly after the given dose, in the range of few days to one week. This corresponds a little bit with the result of Wilkes and Barton-Burke [22], who mentioned that chemotherapeutic agents caused side effects that can appear few days to few weeks after chemotherapy administration. They agree with us in that the side effects may start within one week but differs from us about starting the side effects after few weeks. This may be related to the type of side effect spoken about. Some side effects physiologically appear earlier than others.

On the other hand, upon patients` satisfaction after chemotherapy, unfortunately, only one-quarter of the studied patients felt better after chemotherapy. This became in contrast to Adenipekun and Soyannwo [23], who found that the majority of their studied patients coped with side effects of chemotherapy and were satisfied with the care provided. This may be due to the shortage of the number of nurses working in this only and central hospital for cancer in the Gaza Strip, which is overcrowded with cases, which decreases the nurse/patient contact period. So, nurses' work became limited to giving the chemotherapeutic agents to the patient, with no time left for talking to them, reassuring them, answering their questions, or sharing information with them.

In our study, we found that more than half of the studied patients had a poor total level of knowledge. This may be due to the low educational level of the majority of them or may be due to the lack of continuous educational programs for awareness of the patient. Similar results have been reported by Aziz [24], who found that there is a poor level of total knowledge of the studied patients regarding their disease and its treatment, and there were highly statistically significant differences in patient's level of knowledge about cancer in relation to many items, for example, definition, causes, treatment, and purpose of chemotherapy.

As regards to physical conditions among patients in the present study, we found that the most common problem was of the gastro-intestinal system, including loss of appetite, nausea, and vomiting, and change in the sense of taste. This finding was supported by the American Cancer Society [25], which reported that chemotherapeutic agents irritate the lining of the stomach and the first part of the small intestine (duodenum), which stimulates certain nerves communicating with the vomiting centre in the brain resulting in nausea and vomiting.

Another finding of this study revealed that the majority of the studied patients had extreme fatigue and a general weakness that affects their daily living activity. This result is supported by Mahoney et al. [26], who reported that there are musculoskeletal changes among patients receiving chemotherapy.

Also, the present study finds that the most common side effects of chemotherapy are fatigue and neutropenia, related to bone marrow suppression. For this reason, the authors believe that the side effects of chemotherapy may negatively affect the daily activity of cancer patients undergoing chemotherapy programs and thus distract them from daily physical activities.

Parallel to this finding is that of Piamjariyakul et al. [27], who reported that helping cancer patients coping with side effects of chemotherapy and providing education and information on care are necessary. Studied patients were independent in the efficacy of basic daily living activities like walking, dressing, feeding, toileting, and instrumental daily living activity like the ability to use telephone, and responsibility of their medication, while more than half of them were dependent on others in bathing, also one-quarter of them were dependent in food preparation and housekeeping.

This result is consistent with the study of Alptekin et al. [28], who reported that the majority of caregivers were living with the patient in the same house and confirmed that the patient needs assistance in one or more of the daily living activities. This may be due to the mean age of the study group, which was 48 years, and as we know that the functional status of the body decreases with increasing age; the self-care ability decreases as well, which in turn may affect the level of independence among patients, so increasing the need for assistance in performing their daily living in an efficient way.

The majority of cancer patients had a low physical, psychological, and social well-being. This may be due to a low level of knowledge about the side effects of chemotherapy and its management, also due to decreased contact with patients “patient comes to outpatient and stay very minimal time than needed in the clinic, because of the overload of cases in the clinic.” This finding is in accordance with Janelsins et al. [29], who suggested that cognitive difficulties in cancer patients receiving chemotherapy are due to the effect of cancer treatment on patient needs. Therefore, the authors believe that the role of nursing was important in our study by providing and improving the physical and psychosocial and spiritual needs of the patient while receiving chemotherapy.

Concerning the relations between the total level of patients‘ knowledge and sociodemographic data, this study revealed that there were highly statistically significant relations between the patients‘ knowledge and their educational level, age, income, and profession. This finding is parallel to Aziz's finding [24], who found that there were statistically significant relations between age and sex and the total knowledge about the disease and its management. As well, there was a highly statistically significant relation between the level of education and total knowledge scores.

The result of the present study revealed that there are highly statistically significant relation between social need and their education level, marital status, and monthly income. There are statistically significant relations between social need and their age, cost of treatment, job, the presence of a “depending-on” person. In contrast, there is a nonstatistically significant relation between social need and gender.

This finding is in the same line with that of Aziz [24], who found that there were statistically significant relations between the social needs of patients in the study and their sociodemographics in age, occupation, the presence of “depending-on” people.

This study revealed that there are highly statistically significant relations between psychological need and education level. Also, there are statistically significant relations between psychological need and age, marital status, cost of treatment, and monthly income. However, there are nonstatistically significant relations between psychological need and sex, job, and the “depending-on” person. In relation to psychological need, [30] shows that unmarried patients with a chemotherapy treatment have poorer survival prospects, “e.g., higher cancer mortality” compared to the married over the last four decades.

This study stated that there are highly statistically significant relations between spiritual need and their education level, cost of treatment, and monthly income. Also, there are statistically significant relations between spiritual need and age, sex, job, and the presence of “depending-on” person. There are nonstatistically significant relations between spiritual need and marital status.

On the other hand, Kandasamy, Chaturvedi et al. [31] suggested that spiritual well-being is an important component of health promotion and coping with the illness of cancer patients and is closely related to the physical and psychological symptoms of distress and they added that it should be addressed appropriately and adequately in a palliative setting.

Patient education and counseling can help reduce stress, improve coping skills, and can contribute to an improvement of the patients' health, which remains a high priority, especially in patients with metastatic disease. Inadequate health literacy, nonadherence, noncompliance, and other barriers present challenges to providing effective patient education [31].

## 5. Conclusion

Our study showed that more than half of the studied patients had poor knowledge about cancer, chemotherapy, and the level of self-care efficacy, regarding the loss of appetite, nausea, vomiting, bleeding, fatigue, hair loss, and dryness of the skin. On the other hand, they had a good level of self-care efficacy related to diarrhea and constipation. While regarding physical problems, the most affected systems were the gastrointestinal tract and dermatological system. Also, the majority of patients were independent in walking, dressing, toileting, and feeding, but more than two-thirds were in need of assistance to handle finance, shopping, housekeeping, food preparation, and travelling. In relation to psychological, social, and spiritual needs, the majority of our studied patients need financial support for treatment, which affects their mental status deeply. They also need support for the improvement of self-image and coping with illness, as they lack reassurance and encouragement.

## 6. Recommendations

  Educating cancer patients and their families on common symptoms of cancer and adverse effects of chemotherapy treatment and providing them with a simplified and comprehensive Arabic booklet including information about cancer and chemotherapy and strategies for management.
  Follow-up care should be available for patients with cancer through phone calls.
  Cooperation of multidisciplinary health team members and levels of involvement of both patients and family members on home care is essential to maintain continuity of patient care.
  Further research is needed to focus on studying factors affecting self-care efficacy for cancer patients.

## Figures and Tables

**Table 1:** Baseline characteristics among the study patients.

Sociodemographic variables	N	%
Age		
From 20 to less than 34	55	36.7
From 35 to less than 50	37	24.7
From 50 to 60	58	38.7^∗^
Mean ± SD 42.67 ± 7.29		
Sex		
Male	68	45.3
Female	82	54.7^∗^
Marital status		
Single	9	6.0
Married	116	77.3^∗^
Widow	20	13.3
Divorced	5	3.3
Job		
Not work	97	64.7^∗^
Work	53	35.3
Education		
Illiterate	35	23.3
Reads and writes	22	14.7
Qualified average	49	32.7^∗^
High qualified	44	29.3
Cost		
Health insurance	142	94.7^∗^
Private insurance	8	5.3
M income^∗^		
Not enough for treatment	116	77.3^∗^
Enough to cover the cost of treatment	34	22.7
Family M^∗^		
One	4	2.7
Two	9	6.0
Three	29	19.3
Four and more	105	70.0^∗^
“Depending-on” persons		
Not found	14	9.3
Parents	19	12.7
Brothers	16	10.7
Sons	31	20.7
Wife or husband	70	46.7^∗^

M income^∗^: monthly income as stated by the patient. Family M^∗^: family members. ^∗^indicate the highest value in statistical results.

**Table 2:** Distribution of the present history regarding chemotherapy and side effects of chemotherapy among the study patients.

Items	N	%
Chemotherapy starting		
From a month to less 6 months	16	10.7
From 6 month to less1year	30	20.0
From one year and more	104	69.3^∗^
Names of chemotherapy		
No	87	58.0^∗^
Yes	63	42.0
Number of doses per a month		
Single dose	89	59.3^∗^
Two doses	34	22.7
Three doses	3	2.0
Four doses	24	16.0
Session duration		
Minutes	9	6.0
Hours	123	82.0^∗^
Days	18	12.0
Sides effects of chemotherapy appearance		
Ddirectly from taken dose	91	60.7^∗^
Through one weak from taken session	44	29.3
Through 2 weeks	15	10.0
Continuous these symptoms		
One day	20	13.3
2–3 days	24	16.0
1 weak	30	20.0
2 weeks or more	76	50.7^∗^
Feel better after chemotherapy		
Yes	28	18.7
No	122	81.3^∗^

^∗^indicate the highest value in statistical results.

**Table 3:** Distribution of patients' psychological, social, and religious needs.

Items	No		Yes	
	N	%	N	%
Social needs:				
To not interfere with caring responsibility and home activity	105	70.0	45	30.0
To improve changes in his/her appearance	70	46.7	80	53.3
To not make a financial burden	131	87.3	19	12.7
To not interfere with employment or work	134	89.3	16	10.7
Psychological needs:				
To feel patience and calm	87	58.0	63	42.0
To cope with his/her life	92	61.3	85	38.7
Talk about their problems with family or with friends	81	54.0	96	46.0
Doing exercise that relived stress (hot shower)	122	81.3	28	18.7
Handling anxiety	114	76.0	36	24.0
Spiritual and religious needs:				
To have positive spiritual life changes	14	9.3	136	90.7
To realize the importance of participation in religious activity	44	29.3	106	70.7
To receive enough spiritual support from religious activity	31	20.7	119	79.3
To sense a purpose/aim for life	76	50.7	74	49.3

**Table 4:** Level of independence in Basic Efficacy of Daily Living (ADLs) among study patients.

Items	Completely dependent		Need assistant		Independent	
	N	%	N	%	N	%
Transferring from the seat or bed	0	0.0	28	18.7	122	81.3^∗^
Travelling	1	0.7	104	69.3^∗^	45	30.0
Dressing	0	0.0	33	22.0	117	78.0^∗^
Feeding	0	0.0	6	4.0	144	96.0^∗^
Bathing	0	0.0	69	46.0	81	54.0
Toileting	0	0.0	10	6.7	140	93.3^∗^
Food preparation	33	22.0	112	74.7^∗^	5	3.3
Ability to use telephone	10	6.7	25	16.7	115	76.7^∗^
Shopping	31	20.7	112	74.7^∗^	7	4.7
Housekeeping	45	30.0	103	68.7^∗^	2	1.3
Responsibility of own medication	4	2.7	67	44.7	79	52.7
Handle the finance	10	6.7	109	72.7^∗^	31	20.7

**Table 5:** The relation between the level of knowledge and patients' need.

Items	Total knowledge							
	Good knowledge			Poor knowledge			*t*-test	
	Mean	±	SD	Mean	±	SD	t	*P* value
Physical need	41.48	±	14.92	31.36	±	10.78	4.814	<0.001^∗∗^
Social need	2.69	±	1.13	1.90	±	0.85	4.839	<0.001^∗∗^
Psychological need	2.36	±	1.25	2.10	±	0.96	1.451	0.149
Spiritual need	3.13	±	1.18	2.71	±	1.01	2.372	0.019^∗^
Total needs	49.66	±	16.86	38.07	±	12.47	4.834	<0.001^∗∗^

SD: standard deviation. *t*: *t*-test: nonsig.: >0.05; sig.: ≤0.05^∗^; high sig.: ≤0.001^∗∗^.

**Table 6:** Relation between social needs and their sociodemographic variables.

Sociodemographic variables		Social need			Tests	
		Mean	±	SD	t/*f*	*P* value
Age	From 20 to less than 34	2.564	±	0.996	6.747	0.002^∗^
	From 35 to less than 50	2.378	±	1.063		
	From 50 to 60	1.879	±	1.010		
Sex	Male	2.309	±	1.069	0.584	0.560
	Female	2.207	±	1.051		
Marital status	Single	3.333	±	0.866	5.412	<0.001^∗∗^
	Married	2.276	±	1.043		
	Widower	1.750	±	0.967		
	Divorced	1.800	±	0.447		
Job	Not work	2.062	±	1.029	−3.087	0.002^∗^
	Work	2.604	±	1.025		
Education	Illiterate	1.657	±	1.056	14.526	<0.001^∗∗^
	Reads and writes	1.818	±	0.588		
	Qualified average	2.245	±	0.693		
	High qualified	2.955	±	1.180		
Cost					4.714	0.010^∗^
	Health insurance	2.282	±	1.032		
	Private insurance	2.875	±	0.991		
M income∗	Not enough for treatment	2.034	±	0.884	−5.057	<0.001^∗∗^
	Enough to cover the cost of treatment	3.000	±	1.255		
Residence	North of Gaza Governorate	2.212	±	0.857	−0.253	0.801
	Gaza Governorate	2.210	±	1.109		
	The middle governorate	2.265	±	1.129		
Family M∗	No	2.000	±	0.000	0.932	0.447
	One	1.750	±	0.500		
	Two	2.111	±	0.928		
	Three	2.552	±	0.948		
	Four and more	2.210	±	1.115		
“Depending-on” persons	Not found	2.071	±	0.730	2.508	0.045^∗^
	Parents	2.789	±	1.228		
	Brothers	1.875	±	0.885		
	Sons	2.000	±	0.856		
	Wife or husband	2.343	±	1.128		

M income^∗^: monthly income. Family M^∗^: family members. SD: standard deviation. f/*t*: F-test/*T*-test. Nonsig.: >0.05; sig.: ≤0.05^∗^; high sig.: ≤0.001^∗∗^.

**Table 7:** Correlation between self-care efficacy and daily living activity.

Self-care efficacy		
	R	*P* value
Daily living of activity	0.423	<0.001^∗^

^∗^indicates the high statistical significance between self-care efficacy and daily living activities.

## Data Availability

The data used to support the findings of this study are available from the corresponding author upon request.

## Additional Points

*Limitations of the study*. There are some obstacles faced by the researcher, such as the following: some patients spent longer time than expected for data collection due to their health condition and their psychological condition which affected the time length for data collection. We are also thankful to cancer patients who have agreed to participate in our study. Many thanks to our colleges, the oncology nurses, who collected the data.
